# Determining diet from faeces: Selection of metabarcoding primers for the insectivore Pyrenean desman (*Galemys pyrenaicus*)

**DOI:** 10.1371/journal.pone.0208986

**Published:** 2018-12-14

**Authors:** Amaiur Esnaola, Aitor Arrizabalaga-Escudero, Jorge González-Esteban, Arturo Elosegi, Joxerra Aihartza

**Affiliations:** 1 Department of Zoology and Animal Cell Biology, University of the Basque Country UPV/EHU, Leioa, Basque Country, Spain; 2 DESMA Estudios Ambientales, Sunbila, Navarre, Spain; 3 Department of Plant Biology and Ecology, University of the Basque Country UPV/EHU, Leioa, Basque Country, Spain; Austrian Federal Research Centre for Forests BFW, AUSTRIA

## Abstract

Molecular techniques allow non-invasive dietary studies from faeces, providing an invaluable tool to unveil ecological requirements of endangered or elusive species. They contribute to progress on important issues such as genomics, population genetics, dietary studies or reproductive analyses, essential knowledge for conservation biology. Nevertheless, these techniques require general methods to be tailored to the specific research objectives, as well as to substrate- and species-specific constraints. In this pilot study we test a range of available primers to optimise diet analysis from metabarcoding of faeces of a generalist aquatic insectivore, the endangered Pyrenean desman (*Galemys pyrenaicus*, É. Geoffroy Saint-Hilaire, 1811, Talpidae), as a step to improve the knowledge of the conservation biology of this species. Twenty-four faeces were collected in the field, DNA was extracted from them, and fragments of the standard barcode region (COI) were PCR amplified by using five primer sets (Brandon-Mong, Gillet, Leray, Meusnier and Zeale). PCR outputs were sequenced on the Illumina MiSeq platform, sequences were processed, clustered into OTUs (Operational Taxonomic Units) using UPARSE algorithm and BLASTed against the NCBI database. Although all primer sets successfully amplified their target fragments, they differed considerably in the amounts of sequence reads, rough OTUs, and taxonomically assigned OTUs. Primer sets consistently identified a few abundant prey taxa, probably representing the staple food of the Pyrenean desman. However, they differed in the less common prey groups. Overall, the combination of Gillet and Zeale primer sets were most cost-effective to identify the widest taxonomic range of prey as well as the desman itself, which could be further improved stepwise by adding sequentially the outputs of Leray, Brandon-Mong and Meusnier primers. These results are relevant for the conservation biology of this endangered species as they allow a better characterization of its food and habitat requirements.

## Introduction

The diet of many consumers is difficult to determine: direct observation can be methodologically challenging and visual identification of prey remains in faeces difficult [1], especially in the case of generalist feeders, which prey upon a large variety of species. Consumers rarely forage at random and prey choice can be affected by prey defence and escape strategies, by nutritional quality, or by spatial and temporal distributions of predators and prey, among other factors [2]. New molecular tools such as DNA metabarcoding allow non-invasive studies of diet, as well as assignation of the consumer species that produced a scat, thus preventing identification errors. These new techniques allow analysing the environmental DNA (eDNA) extracted from faeces [3–5] and the identification of both soft- and hard-bodied prey species to species level, what was impossible by means of traditional morphological techniques [6]. Furthermore, DNA metabarcoding facilitates high-resolution dietary analyses further disclosing trophic and habitat requirements of consumers and providing an invaluable tool to unveil food web structures [7], particularly useful for elusive and endangered species [8–11]. Especially, these innovative techniques make diet studies useful for species conservation. For instance, a cost-effective screening of multiple DNA metabarcodes in faeces detected a broad diversity of plants (99 taxa) in the diet of the vulnerable Italian hare (*Lepus corsicanus*), including items that leave no solid remains or that lack diagnostic taxonomic features [12]. Similarly, Schwarz et al. [13] documented dietary differences consistent across site and year in the diet of male and female harbor seals (*Phoca vitulina*), likely affecting on commercial prey such as salmon. Moreover, the foraging ecology of the Alpine long-eared bat *Plecotus macrobullaris* was inferred from the molecular analysis of faeces and from the ecological requirements of prey, which would be impossible by traditional radio-tracking methods [14]. Furthermore, the analysis of the prey consumed showed that the Mediterranean horseshoe bat *Rhinolophus euryale* does not only rely on the habitats where it directly hunts, but also on other habitats of vital importance for its prey’s larval stages, where bats do not forage [15]. Results like these help setting up guidelines for species management.

High throughput sequencing methods are effective when applied to the dietary analyses of predators [16] since they enable the examination of very degraded, fragmented and different DNA pieces without previous knowledge of prey identity [1,11,17]. However, some methodological constraints must be taken into account. On the one hand, DNA quality is affected by its transition time across the gut, as well as the exposure of scats to environmental conditions (e.g. temperature oscillations, sun, rain, humidity and fungal attacks) [2,18,19]. On the other hand, the high sensitivity of molecular methods may produce abundant false positives as a consequence of secondary predation, scavenging, or contamination from any source [20]. Finally, the completeness of diet characterization depends on methodological details such as the taxonomic coverage of primers, the spectrum of consumed prey species, PCR strategy, sequencing workflows, bioinformatics decisions or the information available in databases (e.g. [21–23]).

Studies that target terrestrial animals usually rely on markers within the mitochondrial cytochrome c oxidase subunit I (COI) region, since it has a high copy number and variation suitable for allowing species-level identification [24,25]. Indeed, the COI has one of the most complete reference databases nowadays, particularly well represented for many invertebrate taxa in GenBank (http://www.ncbi.nlm.nih.gov) and BOLD System (http://www.boldsystems.org/). Nevertheless, even though the COI is short enough to be identified in fairly fragmented DNA sequences, for very fragmented sequences such as those usually found in faeces, shorter markers have been used successfully (e.g. [26]). The choice of the markers is usually guided by DNA reference databases, but also depends on the research question [27]. In this sense, Alberdi et al. [21] showed that the results of dietary analyses depend on the selection of marker regions, amplicon sizes, primers, as well as the taxonomic level required. While various primers do detect some prey species, detection of others is primer-dependant. If too long regions are amplified, some species will not be identified, resulting in false negatives. This effect will inflate the difference between samples or individuals, thus yielding skewed information about intraspecific variability. Therefore, the selection of the marker region and primers is a critical decision in any DNA metabarcoding study, as factors such as primer length or specificity have a great effect on the results [24,28].

False positives and false negatives in dietary studies can have strong implications in the interpretation of predator ecology, as well as in ecosystem management [29]. Successful identification of relevant prey taxa is key to obtain sound conclusions about the ecological role, trophic specialization and conservation of any predator—or their consumed prey [13,30]—. Besides, the need for simultaneous identification of diet and predator identity from faeces makes methodological decisions more demanding, as a broader phylogenetic spectrum must be targeted.

Here we conducted a DNA metabarcoding study to compare different primers for non-invasive determination of the diet and the identity of an aquatic predator. In particular, we aimed at assessing how different primer pairs—or their combinations—affect both the characterization of a phylogenetically diverse diet and the predator identity.

**Fig 1:**
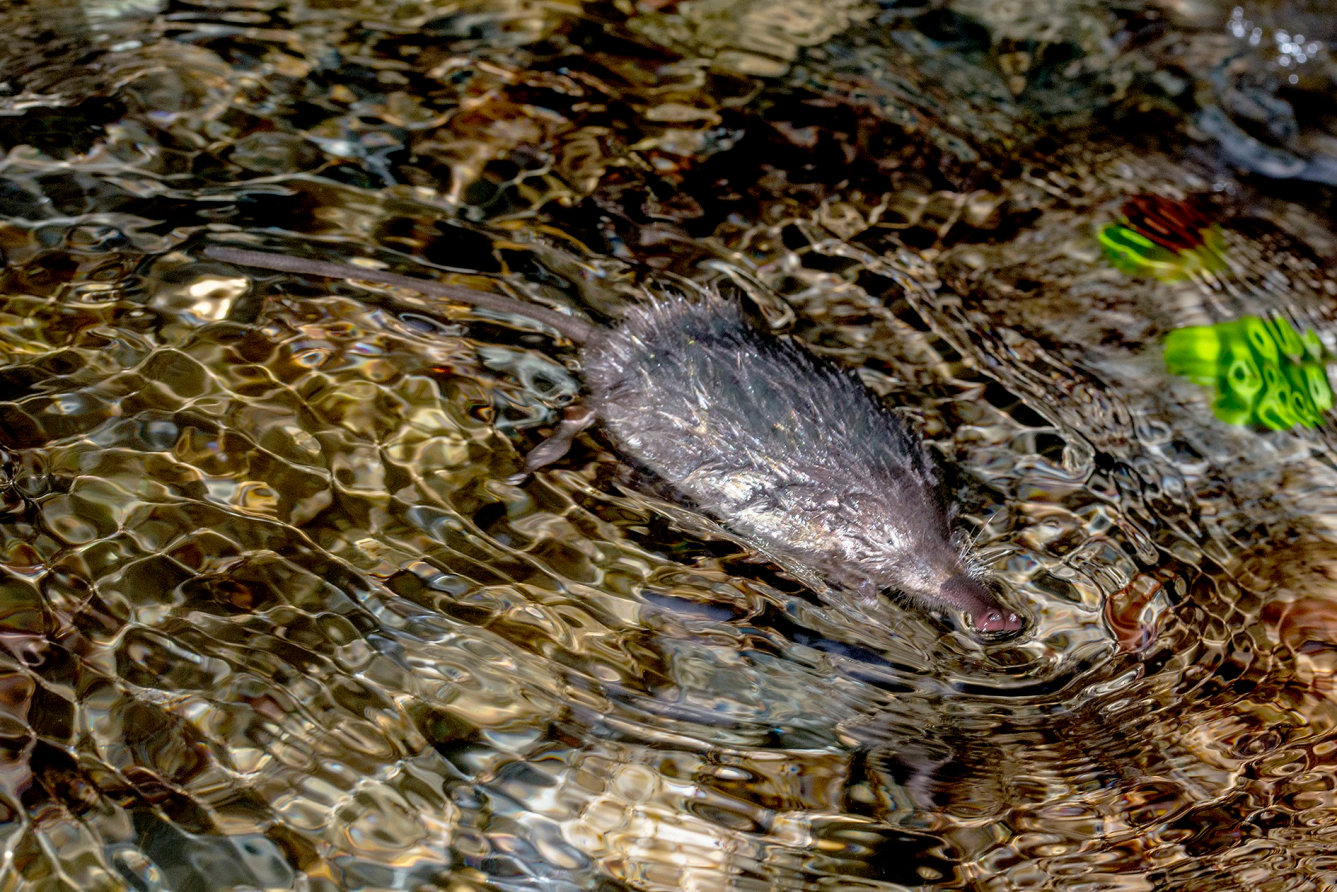
A Pyrenean desman in the Elama Stream. Photo by Joxerra Aihartza.

We chose as a model species the Pyrenean desman (*Galemys pyrenaicus*, É. Geoffroy Saint-Hilaire, 1811, Insectivora, Talpidae) a semi-aquatic insectivorous generalist mammal that lives in cool and clean mountain streams (Fig 1). It is endemic to the northern Iberian Peninsula and the Pyrenees, but its distribution area has been severely reduced during the last decades, being currently listed as Vulnerable in the Red List Categories by the IUCN [31]. Recent research has shown the desman to feed mainly on freshwater invertebrates [32,33] and to prefer riffles to runs or pools [34]. Even so, there is still a lack of information to adequately manage this endangered species [35,36]. Namely, it is still unknown to which extent the diet of desmans depend on prey availability, and which types of prey they select for; moreover, it is still unclear whether desmans’ reported habitat selection within rivers [34] reflects differences in prey availability or other factors. These and other questions ask for detailed diet studies.

Our study provides valuable information about differences in primer efficiency when using DNA metabarcoding in diet studies, offering an overview of the taxonomic coverage provided by each primer set, as well as by their combinations. Moreover, regarding desman ecology, our results will be useful to better understand and compare the output of published studies (e.g. [32,33]), as well as to unveil the habitat requirements of this endangered species. This information is necessary to design and implement specific management actions for its conservation.

## Materials and methods

### Sample collection

We searched for faeces of the Pyrenean desman in the basins or the Urumea and Oria rivers, in the Basque Country (Northern Iberian Peninsula), between November 2015 and April 2016, either by prospecting rock crevices and roots, or by building artificial shelters (Table 1) specially designed for this species [37]. A total of 24 droppings were preserved in 98% ethanol and frozen at -80°C.

**Table 1:** Location of analysed faecal samples.

Basin	River	Number of faeces	Collected in artificial shelters ^a^	Coordinates ^b^
**Urumea**	Asura	3	No	43°08’03”N 1°48’10”W 43°07’46”N 1°47’07”W 43°08’21”N 1°49’39”W
	Ollin	3	No	43°07’44”N 1°51’01”W 43°07’44”N 1°51’01”W 43°07’37”N 1°51’00”W
	Añarbe	3	No	43°13’09”N 1°51’17”W 43°13’18”N 1°51’05”W 43°13’18”N 1°51’05”W
	Elama	9	Yes	43°12’37”N 1°48’38”W 43°12’37”N 1°48’38”W 43°12’37”N 1°48’38”W 43°10’59”N 1°47’59”W 43°10’59”N 1°47’59”W 43°10’58”N 1°47’56”W 43°10’58”N 1°47’56”W 43°10’57”N 1°47’56”W 43°10’57”N 1°47’56”W
**Oria**	Leitzaran	6	Yes	43°08’59”N 1°57’19”W 43°08’59”N 1°57’19”W 43°08’59”N 1°57’19”W 43°08’59”N 1°57’18”W 43°09’00”N 1°57’19”W 43°09’00”N 1°57’19”W

^a^ Indicates whether samples have been collected in artificial shelters (Yes) or elsewhere (No).

^b^ Indicates the exact position of the localities where the samples were collected.

This study is part of a broader research on the spatial and trophic ecology of the Pyrenean desman, which met local legal requirements and was approved by the Ethics Committee for Animal Welfare of the University of the Basque Country (Ref. CEBA/M20/2016/022). No specific permissions were required for the activities carried out in this study, as samples were not invasively collected and did not involve manipulation of endangered or protected species.

### Selection of universal primers

**Fig 2:**
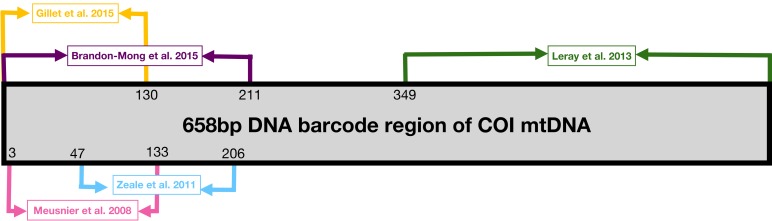
Primer locations. Visual representation of the marker locations in the mitochondrial COI (modified from Brandon-Mong et al. [22]).

We selected five primer sets (Table 2) aiming at a broad taxonomic coverage of potential prey species. These primers amplify fragments of varying lengths within the COI region (Fig 2), currently used as the standard animal barcode region, which has a well-documented reference database.

**Table 2:** Details of the five primer sets used in this study.

Custom name	Primer names	Forward primer(s) (5'-3')	Reverse primer(s) (5'-3')	Length (bp) ^a^	Source
**Brandon-Mong**	**F:**LepF1 **R:**MLepF1_Rev	ATTCAACCAATCATAAAGATATTGG (25bp)	CGTGGAAWGCTATATCWGGTG (21bp)	218	[22]
**Gillet**	**F:**LepF1 (modified) **R:**EPT-long-univR (modified)	ATTCHACDAAYCAYAARGAYATYGG (25 bp)	ACTATAAAARAAAATYTDAYAAADGCRTG (29 bp)	133	[38]
**Leray**	**F:**mlCOIintF jgHCO2198	GGWACWGGWTGAACWGTWTAYCCYCC (26 bp)	TAIACYTCIGGRTGICCRAARAAYCA (26 bp)	313	[39]
**Meusnier**	**F:**Uni-MinibarF1 **R:**Uni-MinibarR1	TCCACTAATCACAARGATATTGGTAC (26 bp)	GAAAATCATAATGAAGGCATGAGC (24 bp)	130	[40]
**Zeale**	**F:**ZBJ-ArtF1c **R:**ZBJ-ArtR2c	AGATATTGGAACWTTATATTTTATTTTTGG (30bp)	WACTAATCAATTWCCAAATCCTCC (24bp)	157	[41]

^a^ Amplicon size excluding primers (bp = base pair).

### DNA extraction, library preparation and NGS sequencing

DNA was extracted using the Qiagen Powerfecal DNA kit (Qiagen Iberia, S.L. Madrid), following the manufacturer guidelines. Subsequently, DNA was PCR amplified from extracts using the five primer sets (Table 2), at the Analytical Services (SGIker) of the University of the Basque Country, UPV/EHU. Samples were purified and a second reaction was performed to index each amplified product and attach Illumina adaptors using the Illumina Nextera v2 kit. Amplifications with Zeale, Gillet, Leray and Meusnier primer sets were performed with the Qiagen Multiplex PCR Kit protocol (Qiagen Iberia, S.L. Madrid, using 12.5 μL Qiagen 2X (1X final), 1.25 μL forward primer (10 μM; 0.5 μM final), 1.25 μL reverse primer (10 μM; 0.5 μM final), 8 μL H_2_O and 2 μL DNA, in a final volume of 25 μL. Amplification with Brandon-Mong primer set was performed with 12.5 μL KAPA HIFI 2X (1X final), 2.5 μL forward primer (10 μM; 1 μM final), 2.5 μL reverse primer (10 μM; 1 μM final), 5.5 μL H_2_O and 2 μL DNA, in a final volume of 25 μL. Each primer set had its own PCR program, modified from the reference to the used reactive, as indicated in the S1 Table. Once amplified, PCR outputs were sequenced in a *Illumina MiSeq NGS platform* (sequencing of 2x300 bp paired-end reads) with the *MiSeq Reagent Kit v3 (600 cycle)*, following the manufacturer instructions.

### Bioinformatic analyses

Paired-end reads were merged using USEARCH [42,43], demultiplexed by primers, adapter and primer sequences were removed, and reads were quality- and length-filtered using CUTADAPT [44]. Then, singletons were removed and the remaining sequences were screened for chimeras using USEARCH. UPARSE algorithm [45] was used to cluster sequences into Operational Taxonomic Units (OTUs) at a 97% similarity threshold. Finally, Genbank nt database was used to assign taxonomy to OTUs using BLAST (https://blast.ncbi.nlm.nih.gov/Blast.cgi). Species level assignments were performed when query sequences matched reference sequences above 98% similarity and 75% overlap [16]. If query sequences matched more than one species in the database, the hit with the longest alignment length was selected. Besides, as a rule, only hits with e-value below 1e-20 were accepted [46] to make sure that the match did not occur by chance.

Subsequent analyses were performed taking into account the occurrence of identified prey taxa (the number of pellets that a taxon was found in) [47]. Primer outputs were also tested to see whether any of the OTUs built from them could also identify the predator itself, i.e. the Pyrenean desman.

### Data analysis

To study the overall effect of primers on variation in species composition of diet, we performed a permutational multivariate ANOVA using *adonis* with 999 random permutations in *vegan* 2.4–6 package [48] for R version 3.4.3 [49]. A Jaccard distance measure was used to calculate dissimilarities between samples. We performed NMDS in *vegan* 2.4–6 package for R to visualize dissimilarities in species composition among samples. Pairwise differences in species composition between primers were also tested using the function *pairwise*.*perm*.*manova* in package *RVAideMemoire* 0.9-69-3 for R [50]. The variation in species composition within primer sets (i.e. the homogeneity) was also tested using the *betadisper* and *permutest* functions with 999 permutations in package *vegan* 2.4–6 for R [48]. Pairwise differences in homogeneity between primer sets were analysed using Tukey's HSD test in package *vegan* 2.4–6 for R [48].

## Results

**Fig 3:**
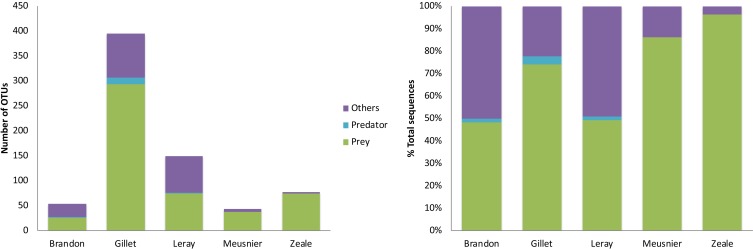
Species-level taxonomic assignment of OTUs obtained for each primer set. Number of OTUs and % of total sequences obtained.

**Fig 4:**
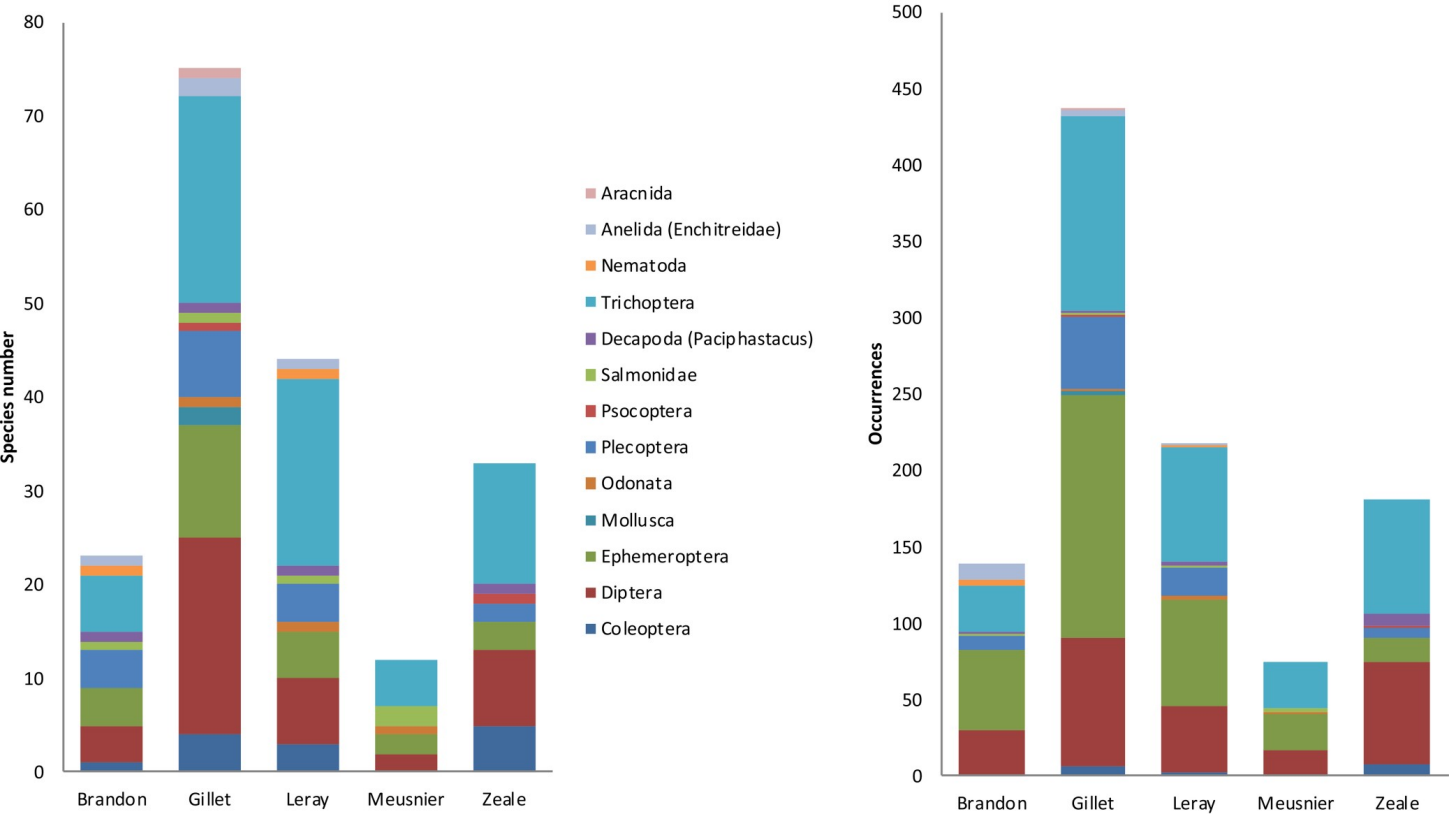
Number of OTUs assigned to species and their occurrences for each primer set. Results taking into account high taxonomical levels.

The sequencing output differed considerably among primer sets, both in the amount of sequence reads and in the number of rough (total) OTUs (Fig 3). Gillet primers yielded the highest numbers of reads and rough OTUs, followed by Leray (S2 Table). Brandon-Mong, Gillet and Leray primers identified the desman itself, but only Gillet and Leray did so in all faecal samples; no other predator whose scats could be mistaken was identified. All five primers identified the most common prey taxa (namely *Baetis* sp., *Hydropsyche* sp., *Odontocerum* sp. and *Psychoda* sp.) but differed considerably in the less abundant prey groups (Fig 4 and S3 Table). Quantitatively, Gillet yielded the largest list of taxonomically assigned OTUs (19.15% of the OTUs), as well as the highest occurrence values. Zeale and Leray primers followed in rough OTUs and assignments (with the 38.5% and the 7.81% of the OTUs assigned, respectively), whilst Brandon-Mong and Meusnier primers showed the lowest efficiency, with less rough OTUs, and only 15.79% and 7.12% of them assigned, respectively. Taking into account the number of taxa assigned, Gillet primer set was the most efficient amplifying DNA of Mollusca, Annelida and arthropods such as Diptera, Ephemeroptera, Plecoptera, Trichoptera or Arachnida (S3 Table); Meusnier primer set was the most efficient amplifying Salmonids, whereas Zeale set was the best amplifying Coleoptera. Although the detected occurrence was lower, Leray primer set was also quite efficient amplifying Trichoptera and Coleoptera. Comparing Gillet with Zeale and Leray separately, Gillet primer set provided more information on Mollusca and arthropods such as Diptera, Ephemeroptera, Plecoptera, Trichoptera and Arachnida than Zeale, whereas the reverse occurred for Coleoptera, Psocoptera and Decapoda. Gillet primer sets outperformed Leray primers for Mollusca, Annelida and arthropods such as Coleoptera, Diptera, Ephemeroptera, Plecoptera, Psocoptera, Trichoptera and Arachnida, while Leray yielded slightly higher values for Nematoda.

**Fig 5:**
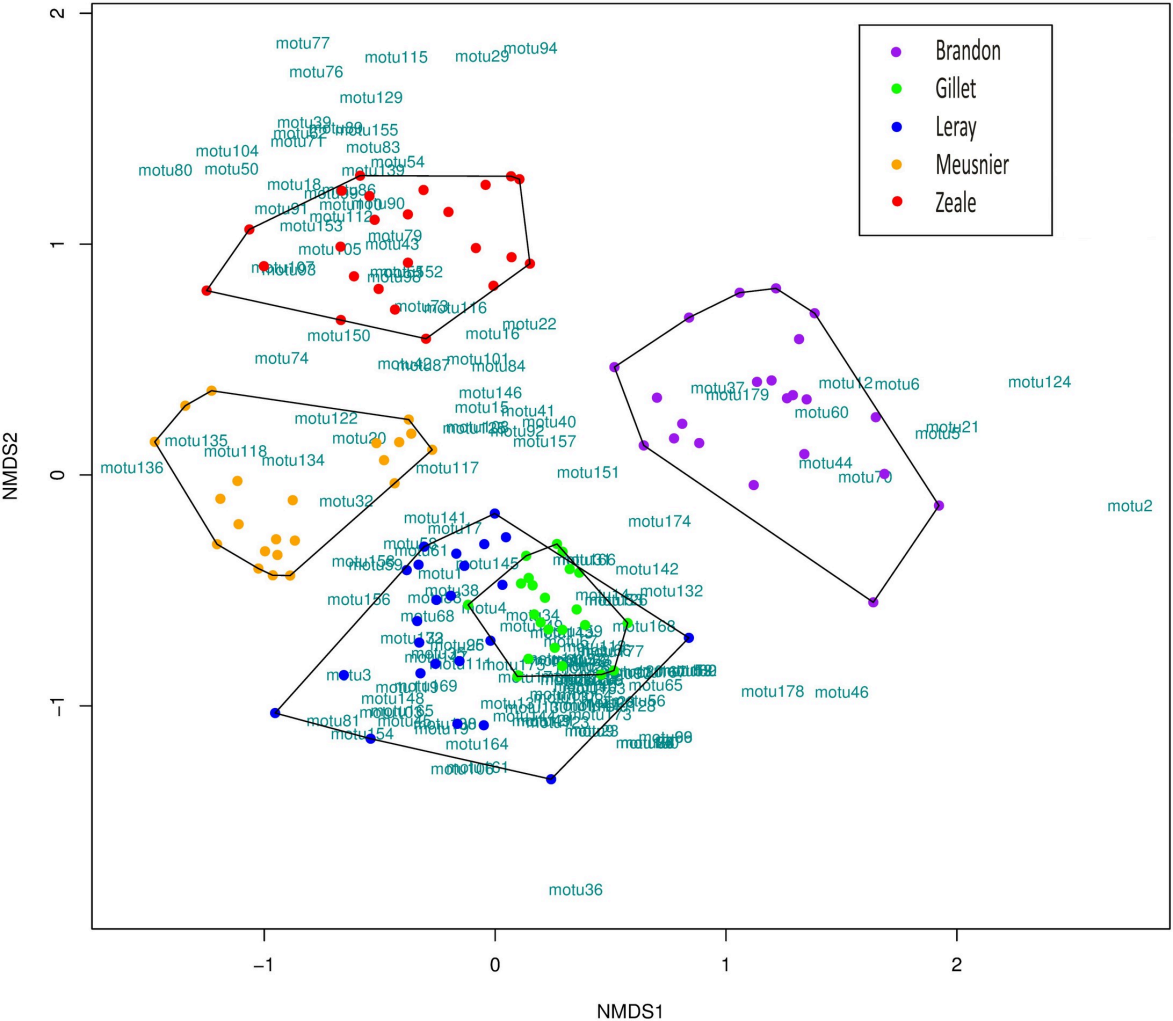
NMDS ordination of samples. Stress = 0.193; k = 2; non-metric fit R^2^ = 0.963. Dots represent individual desman samples and colours different primer sets. OTUs are represented with green letters. More distant dots indicated a more different prey composition.

**Fig 6:**
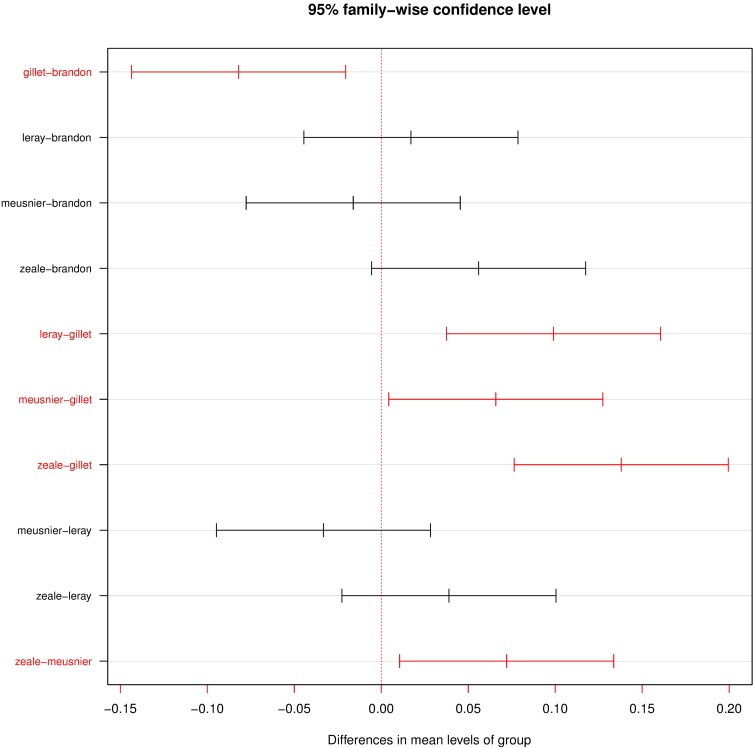
Tukey's HDS plot for all the comparisons between primer sets. Red colour represents significant differences (p < 0.05). The plot shows the differences in mean levels of groups where the largest difference in homogeneity was between Gillet and Zeale, followed by Gillet and Leray.

Primer choice had statistically significant effects on the resulting diet composition (F_(4,115)_ = 15.712; R^2^ = 0.353; p = 0.001). This was clearly illustrated by the NMDS (Fig 5), where samples were clustered in 4 main groups: Zeale, Meusnier, Brandon-Mong and Leray+Gillet. There were significant overall and pairwise differences in species composition between primer sets (overall test: F = 10.425; p < 0.001; pairwise tests: all p = 0.001), but samples amplified with Zeale and Leray or Gillet differed the most in terms of species composition, whereas Leray and Gillet primer sets showed rather similar composition, although Leray seemed to yield higher variability. Moreover, differences in species composition were lowest between Meusnier and Gillet primer sets (p = 0.029), followed by Zeale and Meusnier (p = 0.013) and Gillet and Brandon-Mong (p = 0.003). On the contrary, the highest differences were found between Zeale and Gillet (p < 0.001), followed by Leray and Gillet (p = 0.001) (Fig 6).

**Fig 7:**
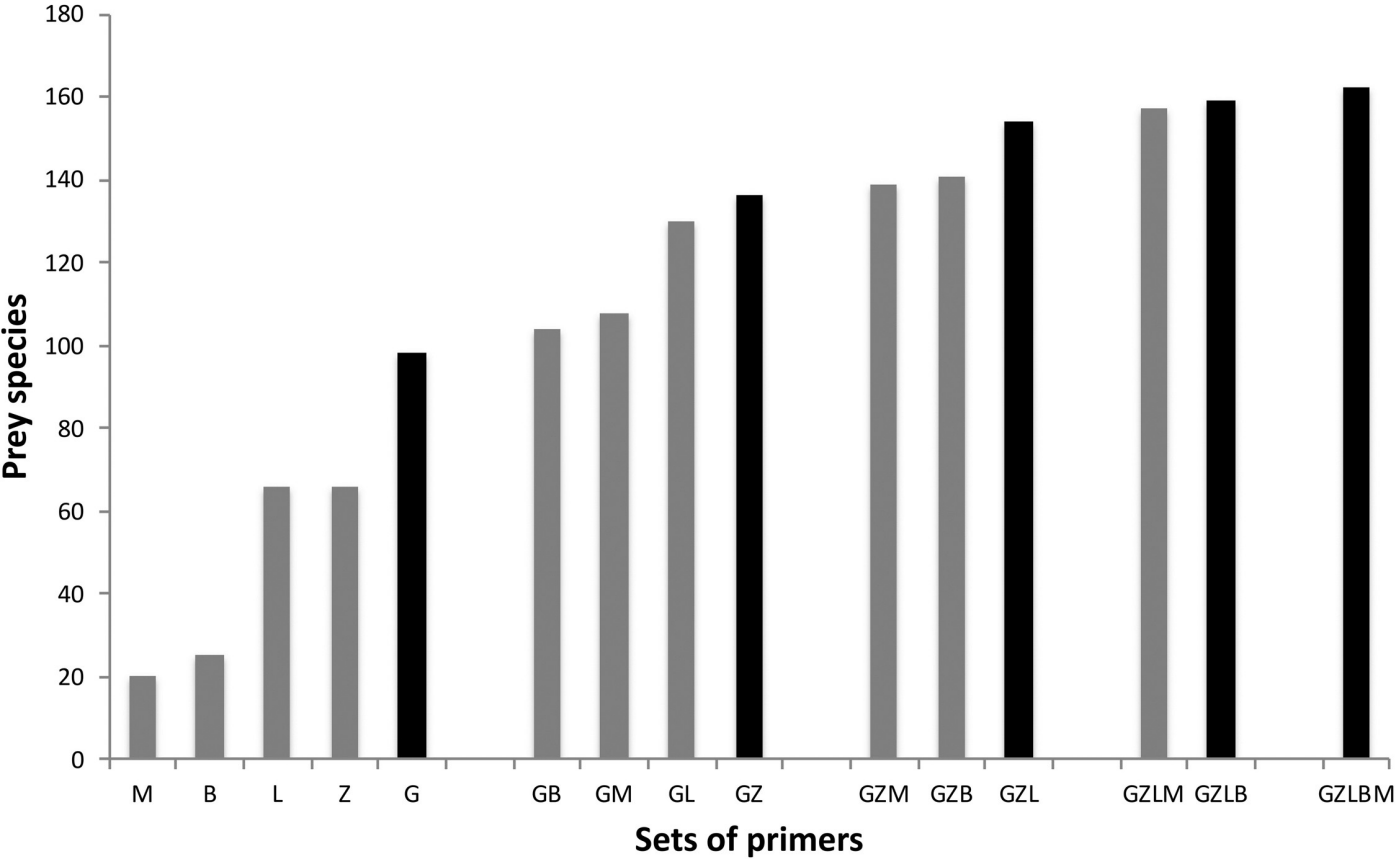
Number of prey species accumulations obtained by different and progressive primer combinations. Progressive primer combinations (one+one, two+one, and so on) were selected according to the highest species accumulation values (black columns) obtained in previous combination-level. B refers to Brandon-Mong primers, G to Gillet, L to Leray, M to Meusnier and Z to Zeale.

We optimized the potential prey species identified by accumulating the amplification outputs of the five primer sets. Gillet provided the highest prey identifications on its own (Fig 7). When combining Gillet with the other sets, the identified species number increased most with Zeale (a total of 129 prey species or accurate taxonomic groups, 37.2% more than only using Gillet). Subsequent combinations yielding the highest increases in species numbers were obtained by adding up Leray first, followed by Brandon-Mong and Meusnier primer sets, in this order, with quantitatively smaller increases in each step.

## Discussion

Our study shows that the selection of molecular markers—even primers targeting different sections of the same gene—considerably affect the characterization of diet. This is particularly important in generalist predators with a phylogenetically diverse diet. Although some prey taxa were consistently detected by all five primer sets used—likely the ones most frequently consumed—, the resulting picture of dietary composition depended on the primer set considered. Our results confirmed those by Alberdi et al. [21] in bats, who had also observed contrasting performance of four primer sets targeting two different genetic markers (the COI and the 16S).

Primer performance differed not only quantitatively, in the total amounts of sequence reads, rough and taxonomically assigned OTUs, and numbers of identified putative prey specific taxa, but also qualitatively, in the proportion of the main phylogenetic groups each primer yielded. These differences may be due to the specific affinity of each primer set to amplify certain taxa, due to the amplicon length, or to the degeneration degree of each primer as well.

Specifically, since longer sequences of DNA resist digestion worse [26], the primer sets targeting longer DNA fragments are less efficient in detecting prey from faeces. The integrity of a sequence of a given length may be related to various factors such as differences in digestion among tissues, the time under digestion, retention time, etc., and this variation differs among prey species (reviewed in [51]). This is partially consistent with our case study, where Gillet primers, targeting almost the shortest fragment of COI gene (Table 1), yielded the highest output in terms of sequence reads, OTUs, and potential prey detected as well. On the contrary, Leray primers, which targeted a similarly broad taxonomic range but with much longer amplicons, retrieved less fragments of DNA and were unable to amplify some taxa that Gillet primer pairs did recover. Nevertheless, primers targeting almost the same stretch length—Gillet and Meusnier, for instance—performed unequally as well, both in terms of quantitative and qualitative data. This may be a consequence of the different degrees of degeneration of the primers [52]. In fact, Gillet and Leray primers, showing the highest degeneration degree (Table 2), are the most successful in terms of OTUs gathered after the PCR, but many of them could not be assigned to any known taxa (only 19.15% and 7.8% of them, respectively), suggesting that they would also amplify many other DNA substrates beyond the targeted COI fragment. On the contrary, primers with lesser degeneration—such as Zeale—produced a much smaller amount of OTUs, but a higher proportion of them (38.5%) were assigned to know taxa, showing a higher bond to the barcode region. Consequently, the varying persistence of relatively long DNA sequences after digestion, and the primers used to amplify them, affect the final perception about the intraspecific variation of individual diets. Therefore, the selection of suitable primer sets for diet analyses is crucial when a wide dietary variation is expected [53].

Recently, a pipeline for the diet analysis of the desman has been published [33], where DNA metabarcoding of old and fresh faecal samples was implemented with nested PCRs. This methodological contribution adds a pre-amplification step to increase the number of reads corresponding to the target taxa. Nevertheless, authors pointed out that their procedure could lead to an increase in the specificity of the amplification, thus losing some essential prey sequences. Our results suggest that such nested PCR would further increase primer bias, multiplying the effect of the two primer sets used. Hawlitschek et al. [33] also proposed that primer cocktails or pools of amplification products of more than one primer pair should be used in future studies to improve the amplification success of the target group. That is precisely what the present work tested and showed to be true.

Regarding the desman trophic ecology, two recent metabarcoding studies provided new data: Biffi et al. [32], employing only the Gillet primer set, described the desman as “more generalist than previously thought” as a consequence of its diverse summer diet, mostly based on Ephemeroptera and Plecoptera in the North of Pyrenees; Hawlitschek *et al*. [33], working in the north-western Iberian Peninsula with Meusnier primers and nested PCR, found that Ephemeroptera and Diptera were the most abundant prey groups. Our data, although carried out in a different area and more limited in sample size, also identified these taxa as relevant components of the desman winter diet. However, the number of species and their occurrence rates largely depended on the primer set considered. To which extent do the results of these studies show methodological differences rather than regional variations in desman prey availability? We can hardly tell. Our results suggest the characterization of the diet of desmans—or any other animal—using a single primer set to be prone to serious biases. These will weaken ecological conclusions such as predator-prey interactions or prey selection patterns, which may be key for implementing conservation measures.

A species might be characterised as generalist due to the overall consideration of many individual-level specialist diets [54] or due to the capacity of individuals to forage on a wide diversity of food items [55]. When analysing the diet of a generalist predator, the choice of primer sets may condition the interpretation of its trophic ecology and specialization. The way foraging habits are perceived can in turn affect the interpretation of community dynamics and ecosystem functioning [56], and thus, any biased description of diet can lead managers to misinterpret food chain structures and to take wrong conservation decisions [57,58]. For instance, trophic relationships have been described with molecular techniques for bats [59–61], birds [62,63], rodents [64] and invertebrates such as snails [65], among others. I t is essential to invest in accurate trophic and spatial ecology studies to obtain detailed knowledge about the trophic requirements of a species. Our results stress the importance of combining different primer sets to detect the widest range of potential prey species and to avoid losing essential information. Methodological decisions affect the assessment of trophic requirements of any animal, as well as management measures based on this assessment [66].

Additionally, when working with endangered and elusive animals it is essential to unambiguously identify the source of faecal samples collected. So far, molecular tools have been useful to provide accurate dietary description from scats in a wide range of predators such as bats [46,67], carnivores [68,69], pinnipeds [8], birds [70] and the desman itself [32]. In the present study desman was identified from faeces with 3 of the tested primer sets, namely Leray, Gillet and Meusnier. However, only Leray and Gillet primer sets successfully identified the desman in all analysed faecal samples, confirming the lower performance of Meusnier to identify Chordata [40]. In general, in diet studies involving insectivorous predators with non-unequivocally identifiable faeces, at least one of the selected primers should be able to amplify DNA of the predator, reducing identification mistakes.

Our results suggest that the diet of Pyrenean desman can be characterised by combining multiple primers, but primer selection must also consider data accuracy and costs. In terms of cost-effectiveness, Gillet and Zeale primer sets would be the best combination to identify the widest taxonomic range of prey, as well as the desman itself. This combination would further improve stepwise by sequentially adding Leray, Brandon-Mong and Meusnier primers, from highest to lowest enrichment. Nevertheless, it must also be taken into account that to combine primers the sequences they amplify must not differ too much in length, to avoid biases towards the smallest amplicons in some NGS sequencing procedures. Thus, in our case the combination of Gillet and Zeale primers would likely result in less problems than the combination of Gillet and Leray, as fragment amplicons with Leray are double in size.

Overall, the present study shows that using different sub-regions in a specific marker yields contrasting results, and highlights the ecological relevance of considering several primer sets to characterize the prey spectrum of generalist predators. In the end, when taking conservation measures for threatened species the methodological procedure could be crucial. For instance, the diet of an endangered animal yields key information about its ecological role and its biology, information that can be essential to protect it. Thus, in diet metabarcoding studies it is necessary to test and assess different primer combinations. Trophic and habitat requirements are key factors in the conservation of many endangered species [71, 72]. Given that results on diet depend so much on the combination of primers used, testing and assessing different primer combinations is necessary in diet metabarcoding studies.

## Supporting information

S1 Table Details of PCR conditions for the five primer sets. (ZIP)

S2 Table Gross sequence reads, sieved sequence reads and number of OTUs obtained with each primer set. (ZIP)

S3 Table Number of species identified (N.Sp.) number of occurrences (Occ.) in each taxonomic group, for each primer set. (ZIP)
