# Supplementary material for: Determining diet from faeces: Selection of metabarcoding primers for the insectivore Pyrenean desman (Galemys pyrenaicus)
Source: PLoS One. 2018 Dec 14;13(12):e0208986. doi: 10.1371/journal.pone.0208986 (PMC6294389; doi:10.1371/journal.pone.0208986)
Supplement: S1 Table — (ZIP) [file pone.0208986.s001.zip › S1_Table.doc]

Table S1. Details of PCR conditions for the five primer sets.

| **PCR stages** | **Zeale** | **Gillet** | **Leray** | **Meusnier** | **Brandon-Mong** |
| --- | --- | --- | --- | --- | --- |
| **Initialization** | 95 °C, 10min | 95ºC, 15min | 95ºC, 10min | 95ºC, 10min | 95ºC, 2min |
| **Denaturation** | 16 cycles:  95 °C, 30 seg  61 °C, 30 seg  72 °C, 30 seg | 40 cycles:  94 °C, 30 seg  45 °C, 45seg  72 °C, 30 seg | 16 cycles:  95 °C, 10 seg  62 °C (-1 °C per cycle), 30 seg  72 °C, 60 seg | 5 cycles:  95 °C, 1min  46 °C, 1min  72 °C, 30 seg | 25 cycles:  95 °C, 15s eg  45 °C, 30 seg  72 °C, 3 min |
| **Annealing** | 24 cycles:  95 °C, 30 seg  53 °C, 30 seg  72 °C, 30 seg |  | 25 cycles:  95 °C, 10 seg  46 °C, 30 seg  72 °C, 60 seg | 35 cycles:  95 °C, 1 min  53 °C, 1 min  72 °C, 30 seg |  |
| **Extension** | 72 °C, 10 min | 72 °C, 10 min | 72 °C, 10 min | 72 °C, 10 min | 72 °C, 10 min |
