# Supplementary material for: Determining diet from faeces: Selection of metabarcoding primers for the insectivore Pyrenean desman (Galemys pyrenaicus)
Source: PLoS One. 2018 Dec 14;13(12):e0208986. doi: 10.1371/journal.pone.0208986 (PMC6294389; doi:10.1371/journal.pone.0208986)
Supplement: S2 Table — (ZIP) [file pone.0208986.s002.zip › S2_Table.doc]

Table S2. Gross sequence reads, sieved sequence reads and number of OTUs obtained with each primer set.

|  | **Brandon-Mong** | **Gillet** | **Leray** | **Meusnier** | **Zeale** |
| --- | --- | --- | --- | --- | --- |
| **Gross sequence reads** | 3754874 | 12667575 | 4364497 | 5050701 | 3110300 |
| **Sieved sequence reads** | 557978 | 11788479 | 1668749 | 4120090 | 2231688 |
| **Rough (Total) OTUs** | 342 | 2063 | 1920 | 604 | 200 |
| **Taxonomically assigned OTUs** | 54 | 395 | 150 | 43 | 77 |
