# Supplementary material for: Determining diet from faeces: Selection of metabarcoding primers for the insectivore Pyrenean desman (Galemys pyrenaicus)
Source: PLoS One. 2018 Dec 14;13(12):e0208986. doi: 10.1371/journal.pone.0208986 (PMC6294389; doi:10.1371/journal.pone.0208986)
Supplement: S3 Table — (ZIP) [file pone.0208986.s003.zip › S3_Table.doc]

Table S3. Number of species identified (N.Sp.) number of occurrences (Occ.) in each taxonomic group, for each primer set.

|  | **Brandon-Mong** | | **Gillet** | | **Leray** | | **Meusnier** | | **Zeale** | |
| --- | --- | --- | --- | --- | --- | --- | --- | --- | --- | --- |
| **Taxonomic group** | **N.Sp.** | **Occ.** | **N.Sp.** | **Occ.** | **N.Sp.** | **Occ.** | **N.Sp.** | **Occ.** | **N.Sp.** | **Occ.** |
| Annelida (*Enchitreidae*) | 1 | 10 | 2 | 4 | 1 | 1 | 0 | 0 | 0 | 0 |
| Arachnida | 0 | 0 | 1 | 1 | 0 | 0 | 0 | 0 | 0 | 0 |
| Coleoptera | 1 | 1 | 4 | 7 | 3 | 3 | 0 | 0 | 5 | 8 |
| Decapoda (*Paciphastacus*) | 1 | 1 | 1 | 2 | 1 | 2 | 0 | 0 | 1 | 7 |
| Diptera | 4 | 29 | 21 | 84 | 7 | 43 | 2 | 17 | 8 | 67 |
| Ephemeroptera | 4 | 53 | 12 | 159 | 5 | 70 | 2 | 24 | 3 | 15 |
| Lepidoptera | 1 | 1 | 21 | 39 | 20 | 51 | 8 | 25 | 32 | 135 |
| Mollusca | 0 | 0 | 2 | 2 | 0 | 0 | 0 | 0 | 0 | 0 |
| Nematoda | 1 | 4 | 0 | 0 | 1 | 1 | 0 | 0 | 0 | 0 |
| Odonata | 0 | 0 | 1 | 2 | 1 | 2 | 1 | 1 | 0 | 0 |
| Plecoptera | 4 | 9 | 7 | 47 | 4 | 19 | 0 | 0 | 2 | 7 |
| Psocoptera | 0 | 0 | 1 | 1 | 0 | 0 | 0 | 0 | 1 | 2 |
| Salmonidae | 1 | 1 | 1 | 1 | 1 | 1 | 2 | 2 | 0 | 0 |
| Trichoptera | 6 | 31 | 22 | 127 | 20 | 75 | 5 | 31 | 13 | 75 |
| **Total** | **24** | **140** | **96** | **476** | **64** | **268** | **20** | **100** | **65** | **316** |
